# Consensus reporting guidelines to address gaps in descriptions of ultra-rare genetic conditions

**DOI:** 10.1038/s41525-024-00408-w

**Published:** 2024-04-06

**Authors:** Ali AlMail, Ahmed Jamjoom, Amy Pan, Min Yi Feng, Vann Chau, Alissa M. D’Gama, Katherine Howell, Nicole S. Y. Liang, Amy McTague, Annapurna Poduri, Kimberly Wiltrout, IPCHiP Executive Committee, Anne S. Bassett, John Christodoulou, Lucie Dupuis, Peter Gill, Tess Levy, Paige Siper, Zornitza Stark, Jacob A. S. Vorstman, Catherine Diskin, Natalie Jewitt, Danielle Baribeau, Gregory Costain

**Affiliations:** 1 Temerty Faculty of Medicine, University of Toronto, Toronto, ON Canada; 2 Program in Genetics & Genome Biology, SickKids Research Institute, Toronto, ON Canada; 3 Department of Paediatrics, University of Toronto, Toronto, ON Canada; 4 Department of Pediatrics, King Abdulaziz University, Jeddah, Saudi Arabia; 5 Department of Molecular Genetics, University of Toronto, Toronto, ON Canada; 6 Division of Neurology, Hospital for Sick Children, Toronto, ON Canada; 7 Department of Neurology, Boston Children’s Hospital, Boston, MA USA; 8 Division of Newborn Medicine, Department of Pediatrics, Boston Children’s Hospital, Boston, MA USA; 9 Department of Pediatrics, Harvard Medical School, Boston, MA USA; 10 Department of Neurology, Royal Children’s Hospital, Melbourne, VIC Australia; 11 Murdoch Children’s Research Institute, Melbourne, VIC Australia; 12 Division of Clinical and Metabolic Genetics, Hospital for Sick Children, Toronto, ON Canada; 13 Department of Neurology, Great Ormond Street Hospital, London, UK; 14 Developmental Neurosciences, UCL Great Ormond Street Institute of Child Health, London, UK; 15 Department of Neurology, Harvard Medical School, Boston, MA USA; 16 Department of Psychiatry, University of Toronto, Toronto, ON Canada; 17 Division of Psychiatry, Ichan School of Medicine at Mount Sinai, New York City, NY USA; 18 Department of Paediatrics, University of Melbourne, Melbourne, VIC Australia; 19 Victorian Clinical Genetics Service, Melbourne, VIC Australia; 20 Department of Psychiatry, Hospital for Sick Children, Toronto, ON Canada; 21 Autism Research Centre, Holland Bloorview Kids Rehabilitation Hospital, Toronto, ON Canada

**Keywords:** Genetics research, Paediatric research

## Abstract

Genome-wide sequencing and genetic matchmaker services are propelling a new era of genotype-driven ascertainment of novel genetic conditions. The degree to which reported phenotype data in discovery-focused studies address informational priorities for clinicians and families is unclear. We identified reports published from 2017 to 2021 in 10 genetics journals of novel Mendelian disorders. We adjudicated the quality and detail of the phenotype data via 46 questions pertaining to six priority domains: (I) Development, cognition, and mental health; (II) Feeding and growth; (III) Medication use and treatment history; (IV) Pain, sleep, and quality of life; (V) Adulthood; and (VI) Epilepsy. For a subset of articles, all subsequent published follow-up case descriptions were identified and assessed in a similar manner. A modified Delphi approach was used to develop consensus reporting guidelines, with input from content experts across four countries. In total, 200 of 3243 screened publications met inclusion criteria. Relevant phenotypic details across each of the 6 domains were rated superficial or deficient in >87% of papers. For example, less than 10% of publications provided details regarding neuropsychiatric diagnoses and “behavioural issues”, or about the type/nature of feeding problems. Follow-up reports (*n* = 95) rarely contributed this additional phenotype data. In summary, phenotype information relevant to clinical management, genetic counselling, and the stated priorities of patients and families is lacking for many newly described genetic diseases. The PHELIX (PHEnotype LIsting fiX) reporting guideline checklists were developed to improve phenotype reporting in the genomic era.

## Introduction

Genome-wide sequencing and genetic matchmaker services have created a new paradigm for Mendelian disorder delineation^1–3^. Compared to prior decades, when syndrome identification was predominantly phenotype driven, there is now an increasing focus on “genomic ascertainment”^4^ [i.e. initially grouping individuals based on genomic variants of interest rather than (typically non-specific) phenotypes] and on generating functional evidence or usage of non-human model systems to support the disease-variant/gene association. Variability in the consistency of phenotyping and describing of findings is problematic. This variability can be exacerbated by individual sites each contributing only a single patient to an international case-series study, or the extraction of phenotype data from laboratory test requisitions. The field is converging around efforts to develop standardize terminology (e.g. Human Phenotype Ontology or HPO^5^, Medical Action Ontology^6^) and machine-readable, interoperable standards for recording and sharing phenotypes (e.g. Phenopacket Schema^7–9^). However, there are yet no well-defined nor broadly accepted minimum standards for phenotype descriptions of putative novel disorders with multisystem manifestations and/or a neurodevelopmental component.

After a first description of a novel Mendelian disorder is published, patients soon thereafter begin to be diagnosed via clinical genome-wide sequencing^10–12^. These individually ultra-rare^13^ conditions are collectively important contributors to the burden of genetic disease in the population^14^. The typical benefits of a molecular genetic diagnosis^13,15^ are attenuated when there is limited information available to inform genotype–phenotype correlation, natural history, prognostication, and anticipatory care. A key consideration in the assessment of ultra-rare conditions for potential “precision therapy” development is the degree to which the patient’s clinical trajectory can be anticipated^16,17^. Families who are among the first to receive a diagnosis of an ultra-rare genetic disorder have endorsed frustration with the perceived lack of information and support^18,19^. Similarly, clinicians face the same informational barrier, which impacts their abilities to care for and counsel patients and their families^20^.

Published expert opinions, survey data, reviews, and data from patient and family focus groups highlight key informational areas germane to the natural history of ultra-rare genetic diseases^9,20–29^. These informational areas have been studied in the context of more common genetic syndromes like Down syndrome, 22q11.2 deletion syndrome, fragile X syndrome, and the RASopathies^30–38^. We assessed the breadth and depth of phenotype reporting in contemporary descriptions of novel Mendelian genetic diseases across six priority domains: (I) Development, cognition, and mental health; (II) Feeding and growth; (III) Medication use and treatment history; (IV) Pain, sleep, and quality of life; (V) Adulthood; and (VI) Epilepsy. We also assessed in a similar manner follow-up reports appearing in the years following an initial report. These findings provided the impetus for, and guided the development of, the proposed new PHELIX (PHEnotype LIsting fiX) reporting guideline checklists, which complement other tools intended to improve phenotyping for rare genetic diseases^5–9,39^.

## Results

### Contemporary descriptions of new syndromes are often lacking in phenotype details

**Fig. 1:**
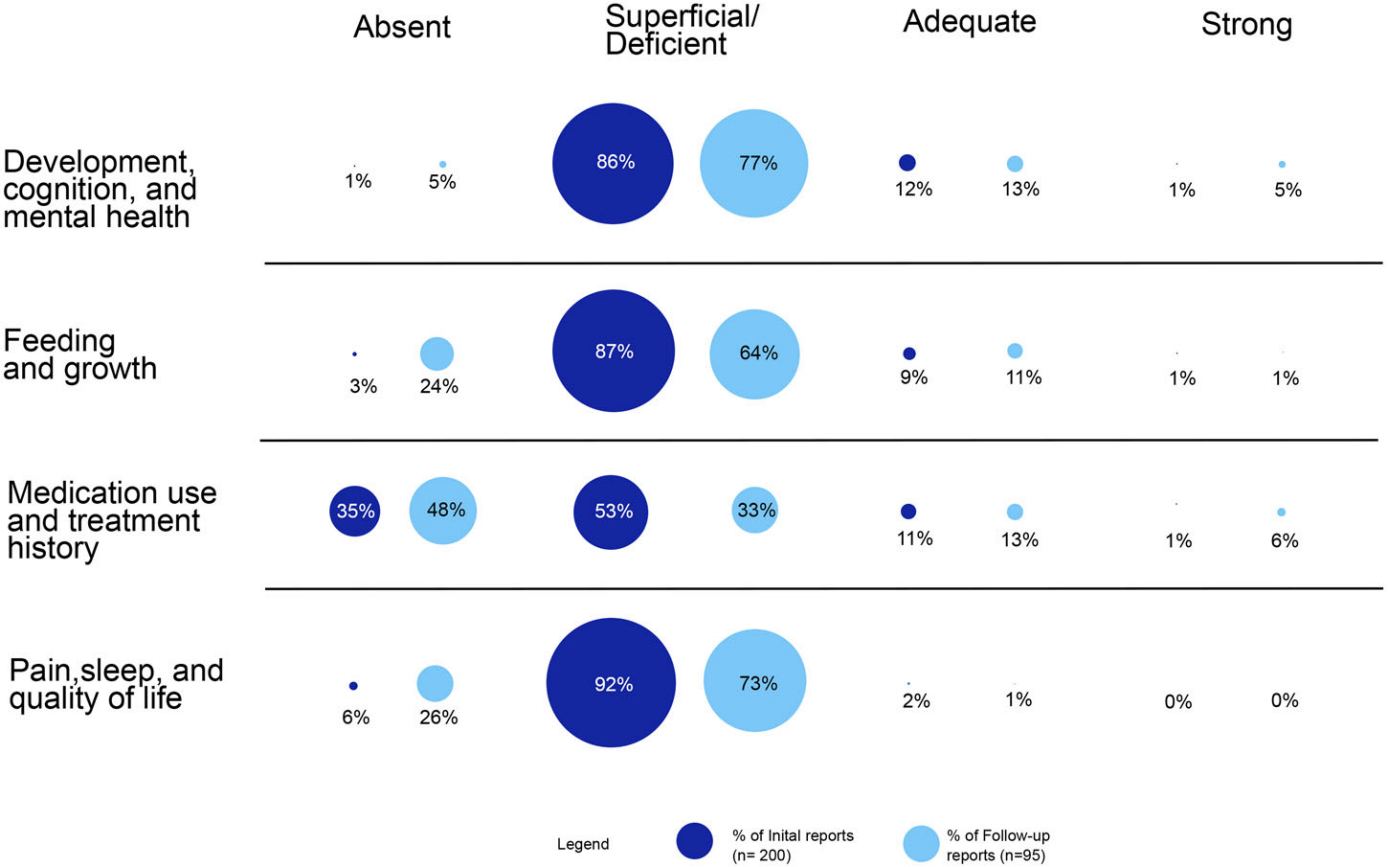
Global qualitative assessments of the reporting of phenotype details germane to Domains I–IV. For each of the four rating categories, the percentage of the initial reports (*n* = 200) are depicted in dark blue and of the follow-up reports (*n* = 95) in light blue. There were no significant differences in the distribution of overall quality ratings between the initial and follow-up reports, for any of the domains (Fisher’s exact tests, *p* > 0.05). See text for details.

The 200 reports of 199 newly discovered genetic disorders included phenotype descriptions for a total of 1856 study participants (median: 7/report, range 2–42). Features of the reports (year and journal of publication) and of the participants (age) are summarized in Supplementary Tables 2 and 3). The overall qualitative assessment of reporting was deemed “superficial/deficient” or “absent” in 87% (Domain I: Development, cognition, and mental health) to 98% (Domain IV: Pain, sleep, and quality of life) of papers (Fig. 1). Five (2.5% of 200) reports were deemed “strong” in any single domain (pertaining to the genetic conditions associated with variants in the genes *ADARB1*^40^, *GNAI1*^41^, *NCAPG2*^42^, *PCDHGC4*^43^, and *SPTBN1*^44^). No reports were deemed “strong” in their reporting across all Domains I–IV. The year and journal of publication were not associated with overall quality assessment of phenotype reporting (data not shown).

Item-specific data supported the overall qualitative assessments of reporting quality (Supplementary Table 3 and Supplementary Figs. 3 and 4). While 97% of papers mentioned developmental concerns in study participants, 21% provided details about cognitive abilities for all the participants and a sole paper^45^ reported results from formal cognitive assessments for all participants (Supplementary Table 4). A common issue was that individuals were identified as having “developmental delay” without further elaboration. Similarly, of the papers that reported neuropsychiatric and behavioural issues in study participants, less than 5% of papers provided details for all participants regarding type/diagnosis, symptom severity, and/or nature of the assessments (Supplementary Table 3). Of the papers that reported on the presence of feeding difficulties, 8% consistently reported on the type/nature of feeding issues and current means of feeding (Supplementary Table 3). Growth parameters at birth were often reported, but 6% of papers reported on two or more growth measurements post-birth to allow for assessment of growth trajectories (Supplementary Table 4). Nearly half of all papers made no mention of participants’ medications or treatment trials, or of the absence thereof. The presence or absence of adverse effects of treatments were explicitly mentioned in just 21% of reports.

Domain V (Adulthood) was assessed in the subset of reports that included at least one adult individual (*n* = 63; adult defined as age ≥18 years) (Supplementary Fig. 1 and Supplementary Table 6). Domain VI (Epilepsy) was assessed in the subset of reports that included at least one study participant with seizures/epilepsy (*n* = 85) (Supplementary Fig. 1 and Supplementary Table 7). Consistent with the findings regarding Domains I–IV, most items were inconsistently or never reported (Supplementary Figs. 3 and 4). For example, papers rarely described proxies for adult functioning such as educational achievement or employment, nor the anti-seizure treatments for individuals with epilepsy.

### Follow-up reports do not consistently address initial gaps in phenotype descriptions

Regarding the 25 genetic conditions first described in 2017, the 95 “follow-up” reports included phenotype descriptions for an additional 334 study participants (median: 1 per report, range 1–25). The overall qualitative assessment of reporting was similarly classified as “absent” or “superficial/deficient” in 81% (Domain III: Medication use and treatment history) to 99% (Domain IV: Pain, sleep, and quality of life) of papers (Fig. 1 and Supplementary Table 4), with no significant differences between the original and the follow-up reports. Eleven reports were deemed “strong” in any single domain (pertaining to the genetic conditions associated with variants in the eight genes *CAMK2A*^46^, *CAMK2B*^46,47^, *DHX30*^48^, *OTUD6B*^49^, *PPP3CA*^50^, *UBTF*^51,52^, *WDR26*^53,54^, and *YY1*^55^). No reports were deemed “strong” in their reporting across each of Domains I–IV. Item-specific data are summarized in Supplementary Table 4.

### Consensus phenotype reporting guidelines

Guideline checklists to enhance the reporting of phenotype data for ultra-rare genetic conditions were developed through a modified Delphi process^56^ and informed by the findings above (Supplementary Table 8). Specifically, items were included based on their superficial/deficient reporting in the literature to date, and on the recommendations of expert collaborators as being data that are both important to capture and feasible to obtain by researchers. The finalized checklist of 33 items across 9 categories is presented in Table 1 (PHELIX_General). To showcase how these guidelines could be expanded over time, additional items specific to epilepsy phenotype reporting are listed in Table 2 (PHELIX_Epilepsy). Extended versions with exemplar references are provided in Supplementary Tables 9 and 10. Examples of common deficiencies in phenotype reporting are provided in Supplementary Table 11.

**Table 1:** Phenotype reporting checklist (PHELIX_General version 1.0)

Phenotype category	Recommendation category	Specific recommendation for inclusion in report
1. Development and cognition	Strongly recommended	i. Standard and specific diagnostic term(s) for cognitive or developmental issue(s) ii. Level of cognitive functioning or degree of developmental delay iii. Age of attaining major milestones iv. Quantitative results from psychometric testing OR explicit acknowledgement that these results were not available
	Optional, but encouraged	v. Narrative summary describing progression/change in cognitive or development issue(s) over time.
2. Behaviour and neuropsychiatric conditions	Strongly recommended	i. Standard and specific diagnostic term(s) for behavioural issues
	Optional, but encouraged	ii. Reported functional impact of behavioural/psychiatric condition iii. Age at diagnosis and/or age at first concern for behavioural issues iv. Impact of treatments/interventions, as reported by individuals, families, and/or clinicians
3. Other medical conditions	Strongly recommended	i. Major medical conditions ii. Presence or absence of issues in the following areas (if potentially associated with the condition under study): • Visual acuity and field of vision • Hearing ability • Speech/communication • Continence/toileting • Ambulation
4. Feeding issues	Strongly recommended	i. Functional impact of feeding issues ii. Current feeding method (e.g. oral, gastrostomy tube)
	Optional, but encouraged	iii. Age at first concern for feeding issues iv. Interventions and supports for feeding issues (e.g. feeding tube support)
5. Growth	Strongly recommended	i. Birth growth measurements AND gestational age-corrected centiles ii. Growth measurements (absolute values and z-scores) at two or more post-birth timepoints (where possible)
6. Medication and treatment history	Optional, but encouraged	i. Details of efficacious treatment(s) ii. Severe adverse events/reactions
7. Pain, sleep, and quality of life	Optional, but encouraged	i. Presence or absence of pain/neuroirritability ii. Presence or absence of abnormal sleep patterns/sleep disturbance iii. Qualitative description of proxies for quality of life, via patient and/or caregiver report iv. Direct assessment of quality of life using established measure(s), via patient and/or caregiver report
8. Indicators of adult functional outcome	Strongly recommended	i. Age at which the adult was last seen/phenotyped ii. Description of educational achievement iii. Nature of any employment (past and/or present)
	Optional, but encouraged	iv. Relationship status (past and/or present) v. Reproductive history
9.Other	Strongly recommended	i. Confirmation of informed consent to participate in the research study and to include the above phenotype information, for each participant ii. Distinguish between “not assessed” and “assessed and not present,” for every aspect of a phenotype described in the report and for each participant iii. Description of how phenotyping was performed (e.g. direct assessment by study team member(s), review of medical records, information provided on testing requisition), for each participant iv. Use of phenotype ontologies (e.g. HPO, ICD-11) and reporting tools (e.g. Phenopackets Schema) to standardize reporting, for each participant v. [For deceased participants] Cause of death

*HPO* Human Phenotype Ontology, *ICD-11* International Classification of Diseases 11th Revision.

**Table 2:** Phenotype reporting recommendations for unprovoked seizures/epilepsy in individuals with newly described multisystem and/or neurodevelopmental Mendelian disorders (PHELIX_Epilepsy version 1.0)

Phenotype subcategory	Recommendation category	Specific recommendation for inclusion in report
1. Epilepsy syndrome and severity	Strongly recommended	i. Seizure type(s) (per ILAE) ii.Age at seizure onset iii. EEG findings (including age(s) at time of study) iv. Epilepsy syndrome(s) (per ILAE) v. Findings that support the diagnosis of the epilepsy syndrome(s) (e.g. specific EEG findings) vi. Seizure frequency at last clinical assessment vii. Qualifiers of overall epilepsy severity (e.g. severe; treatment-refractory)
	Optional, but encouraged	viii. Clarification if EEG data were directly reviewed by members of the study team (versus only report details extracted from medical record) ix. Number of seizures requiring hospitalization in specific timeframe (e.g. last year)
2. Pharmacological interventions	Strongly recommended	i. Current and past medication name(s) ii. Perceived impact on seizure control
	Optional, but encouraged	iii. Dose iv. Duration of treatment trial v. Adverse effects/events due to the intervention
3. Non-pharmacological interventions	Strongly recommended	i. Intervention/procedure details (e.g. ketogenic diet, neurosurgery) ii. Perceived impact on seizure control
	Optional, but encouraged	iii. Adverse effects/events due to the intervention
4. Brain imaging findings	Strongly recommended	i. Brain imaging findings (including age(s) at time of study)
	Optional, but encouraged	ii. Clarification if brain imaging data were directly reviewed by members of the study team (versus only report details extracted from medical record)
5. Other	Optional, but encouraged	i. Narrative summary of the progression of the individual’s seizure(s)/epilepsy phenotype over time ii. Narrative summary of the progression of the individual’s non-epilepsy phenotype over time (e.g. see PHELIX_General guidelines)

*EEG* electroencephalogram, *ILAE* International League Against Epilepsy.

## Discussion

Our results reveal that phenotype information relevant to clinical management, genetic counselling, and the stated priorities of patients and families, is lacking for many newly described genetic diseases. Although most published reports acknowledged the key phenotype domains assessed, few original or follow-up reports included clinically relevant details. To address this issue, we propose reporting guideline checklists for use by researchers and journals. Use of these guidelines could improve phenotype reporting in the era of genotype- and matchmaker service-driven reports of novel syndromes. Decision making about precision genetic or other therapy development, including the potential for N-of-1 trials, may be contingent on our understanding (or lack thereof) of the natural history of a given ultra-rare genetic disease^16,17,57^.

Reasons for under-reporting phenotype data are likely multiple and complex. First, these data may not be readily available to the referring clinician or laboratory collaborators, and “phenotyping is hard,”^58^ especially for older individuals with extensive past histories. Review of lifetime medical records, and/or a brief, targeted interview with patients and/or their caregiver(s), should be sufficient to gather most of the information outlined in Tables 1 and 2. Second, these data may not be requested by the coordinating research team that is leading the publication effort. In our experience, many groups design their own data collection forms that ask for no or only general details regarding issues outside of that group’s specific phenotype(s) of interest. Third, unlike for example DNA sequencing methods, there are no defined minimum reporting standards for phenotyping to guide peer reviewers and journal editors. Finally, there may be a belief that phenotype reporting in initial descriptions of novel genetic diseases is less important than establishing an association between variation in the gene and (any) disease phenotype. Although the hope may be that future reports will then describe many more individuals and include detailed phenotype data, we did not find evidence that this is consistently happening in practice in a timely manner.

We recognize several limitations of our review and guideline development methods. We selected only 10 top-tier genetics journals for our systematic review. The generalizability of our findings to reports published in other specialty-specific or organ system-specific journals is unclear. Out of necessity given the lack of validated tools, we created a new data collection questionnaire to assess the reporting of phenotype data and relied on subjective assessments from raters for some items. We selected broad phenotype domains based on our combined clinical experiences and the published literature; however, these domains are not the only important components of phenotyping. Ours was a paediatrics-focused effort, reflecting the phenotypes that are currently driving most Mendelian gene discovery efforts. Other groups may develop and add-on reporting criteria for additional specific phenotype elements, as we did for epilepsy, and continue to refine the general adult phenotype elements (Table 2). We also restricted our initial focus to cross-sectional reporting, and additional guidance will be needed for evaluating within-individual natural history. Finally, our reporting guidelines have not yet been applied prospectively to assess feasibility and utility.

We propose minimum standards for phenotype descriptions of putative novel disorders with multisystem manifestations and/or a neurodevelopmental component in children. Our intent is to encourage researchers to collect and share more details about key phenotypes where possible, recognizing that such efforts will be more challenging for some research groups and study designs (e.g. laboratory testing-based cohorts) than others. There are many forms of valid scholarship in the descriptions of rare diseases, and we strongly support multi-faceted approaches to phenotype delineation. Further refinement of our proposed reporting guidelines is an important consideration, including by collecting additional input from key stakeholders (e.g. rare disease organizations, journal editors). A key next step is to better integrate technologies for systematic phenotype collection and data sharing^9,59^. Our efforts were intended to be complementary to the Phenopackets Schema, and our findings provide further impetus for sharing phenotypic data in forms that are standardized and computable. Improved reporting of phenotype aspects like craniofacial morphology (dysmorphic features)^60^ and congenital anomalies could help with interpreting variants of uncertain significance and assessing phenotypic “fit”^61,62^. The aim of the PHELIX guideline checklists is to decrease the variability in the consistency of phenotyping and description of findings, and thereby enhance the ongoing clinical care of individuals with genetic conditions.

## Methods

### Systematic review

We utilized DistillerSR Version 2.35 for searching, screening, and data extraction (DistillerSR Inc, 2022; accessed January 2022–January 2023). We identified all first reports of novel genetic conditions discovered through genotype-driven ascertainment that result in multisystem and/or neurodevelopmental phenotypes, which were published in 1 of 10 genetics journals that are known for publishing novel reports of ultra-rare genetic conditions (*American Journal of Human Genetics*, *American Journal of Medical Genetics Part A*, *Clinical Genetics*, *European Journal of Human Genetics*, *Genetics in Medicine*, *Genome Medicine*, *Human Molecular Genetics*, *Journal of Medical Genetics*, *Nature Genetics*, *PLoS Genetics*) during a 5-year period (1 January 2017–31 December 2021). We selected this period because of the rise to prominence of “gene matchmaker” tools in the mid- to late-2010s, and to ensure feasibility of the systematic data extraction. The search executed on January 3, 2022, identified 3243 articles (Supplementary Table 1 and Supplementary Fig. 1). Exclusion criteria were: (i) prenatal or neonatal lethal phenotype, (ii) case report or description of a single family, (iii) new gene for a known clinical syndrome (e.g. Joubert syndrome, Noonan syndrome), (iv) chromosome disorder with non-recurrent breakpoints that did not definitively implicate a specific gene, (v) potential genotype–phenotype expansion rather than a novel disorder. We also excluded large-scale gene discovery efforts in populations with common complex diseases and/or clinical testing laboratory cohort studies, where the a priori expectation for detailed phenotype descriptions was low. After both title and abstract screening and full-text review stages, *n* = 200 reports describing 199 distinct monogenic conditions met the inclusion criteria (two reports of a novel condition were published at the same time; Supplementary Fig. 1). For the subset of 25 genetic conditions first described in 2017, we performed an additional search using DistillerSR on June 1, 2022 (Supplementary Table 1) to identify subsequent published case descriptions in any journal (total *n* = 95; Supplementary Fig. 2). Reference review and additional Internet searching did not identify any other “follow-up” reports.

### Data extraction and analysis

For each published article (*n* = 295), study team members (authors A.A., A.J., A.P., M.Y.F.) adjudicated the phenotype data pertaining to six priority domains [(I) Development, cognition, and mental health; (II) Feeding and growth; (III) Medication use and treatment history; (IV) Pain, sleep, and quality of life; (V) Adulthood; and (VI) Epilepsy] using a custom designed data extraction form (Supplementary Tables 3, 5, and 6). Domains I–VI were included based on the study team’s clinical experience and review of the aforementioned published expert opinions, survey data, reviews, and data from patient and family focus groups^9,20–29^. Data for Domains V and VI were extracted separately from Domains I to IV. The total 46-item form was developed by members of the study team (authors A.A., A.J., C.D., N.J., D.B., G.C.) with clinical expertise in medical genetics, psychiatry, development, general paediatrics, paediatric palliative care, paediatric complex care, and paediatric hospitalist medicine. Each domain was associated with multiple issue-specific items. Descriptive statistics were calculated using Microsoft Excel.

A separate overall qualitative assessment of reporting quality (“strong”, “adequate”, “superficial/deficient”, “absent”, or “not applicable”) was also assigned for Domains I–IV. The overall qualitative assessment of reporting strength in each domain for each report was subjective and informed by data collected in the sub-questions (Supplementary Table 2). For the assessors, “absent” was defined as “no, or almost no, reporting of phenotype information [in this Domain]” and exemplified by reports with >80% of sub-questions having “No” or “Never” responses. “Superficial/deficient” was defined as “modest reporting of phenotype information [in this Domain], often lacking in detail and with concerning gaps from a genetic counselling perspective” and exemplified by reports with 50–80% of sub-questions having “No” or “Never” responses. “Adequate” was defined as “satisfactory reporting of phenotype information [in this Domain], in both breadth and depth, to facilitate genetic counselling and answer initial (common) clinician/family questions” and exemplified by reports with 20–50% of sub-questions having “No” or “Never” responses. “Strong” was defined as “intentional reporting of all available phenotype information [in this Domain], with a breadth and depth that would facilitate genetic counselling and answer initial and follow-up clinician/family questions” and exemplified by reports with <20% of sub-questions having “No” or “Never” responses. For reports with small numbers of participants, the percentage ranges did not apply. We confirmed high inter-rater reliability between the two independent assessors (>80% agreement) based on their blinded review of a subset of the same reports; discordant classifications were discussed together as a group to arrive at a consensus, before the raters proceeded to review the remainder of the reports independently.

### Development of phenotype reporting guidelines through a modified Delphi process

Medical experts from member institutions of the International Precision Child Health Partnership (IPCHiP) participated in a modified Delphi process^56^. IPCHiP institutions included: Murdoch Children’s Research Institute/Royal Children’s Hospital (Melbourne, Australia), The Hospital for Sick Children (SickKids®; Toronto, ON, Canada), University College London/Greater Ormond Street Hospital (London, UK), and Boston Children’s Hospital (MA, USA)^63,64^. At the suggestion of the original study team members, additional expertise was sought in: (i) neuropsychological assessment and cognitive phenotyping (via Seaver Autism Center for Research and Treatment; NY, USA)^65–67^, and (ii) adult phenotyping (via University Health Network; Toronto, ON, Canada)^68,69^. Authors J.C., L.D., P.G., T.L., P.S., Z.S., J.A.S.V., C.D., N.J., and D.B. contributed to the initial refinement of guidelines for Domains I–V. We sent out three electronic surveys to the above authors (minimum engagement rate >50%) over a five-month period to define and prioritize the reporting criteria. We then hosted an online meeting that incorporated independent voting on inclusion/exclusion of each draft item. The meeting was recorded, to allow for asynchronous viewing by those expert volunteers who were unable to attend in real-time. Authors V.C., A.D., K.H., N.S.Y.L., A.T., A.P., and K.W. contributed to the initial refinement of guidelines for Domain VI (Epilepsy). Similarly, we used a series of two electronic surveys to define and prioritize the reporting criteria. All authors reviewed, revised, and ultimately approved the reporting guideline checklists for Domains I–VI reported herein. The guideline checklists will be uploaded to the EQUATOR (Enhancing the QUAlity and Transparency Of health Research) Network website (https://www.equator-network.org/) as the PHELIX_General (Table 1) and the PHELIX_Epilepsy checklists (Table 2).

### Reporting summary

Further information on research design is available in the Nature Research Reporting Summary linked to this article.

### Supplementary information

Supplementary Info

REPORTING SUMMARY

## Data Availability

The datasets analysed during the current study are available from the corresponding authors on reasonable request.
