# Supplementary material for: Consensus reporting guidelines to address gaps in descriptions of ultra-rare genetic conditions
Source: NPJ Genom Med. 2024 Apr 6;9:27. doi: 10.1038/s41525-024-00408-w (PMC10998895; doi:10.1038/s41525-024-00408-w)
Supplement: Supplementary file 1 — Supplementary Info [file 41525_2024_408_MOESM1_ESM.pdf]

SUPPLEMENTARY FILE FOR: Gaps in the phenotype descriptions of ultra-rare genetic conditions: review and multi-center consensus reporting guidelines

TABLE OF CONTENTS

Supplementary Figure 1……………………………………………………...………………2
Supplementary Figure 2……………………………………………………...……………3
Supplementary Figure 3……………………………………………………...…………4
Supplementary Figure 4……………………………………………………...………5
Supplementary Table 1………………………………………………………...……6
Supplementary Table 2……………………………………………………….....…7
Supplementary Table 3……………………………………………………….…...8
Supplementary Table 4……………………………………………………………9
Supplementary Table 5…………………………………………………………..14
Supplementary Table 6……………………………………………………….…15
Supplementary Table 7…………………………………………………………..17
Supplementary Table 8…………………………………………………………..19
Supplementary Table 9…………………………………………………………..20
Supplementary Table 10…………………………………………………………24
Supplementary Table 11…………………………………………………………26
Supplementary Information……………………………………………………...27

Supplementary Figure 1. PRISMA flow diagram for primary search.
See main text for details.

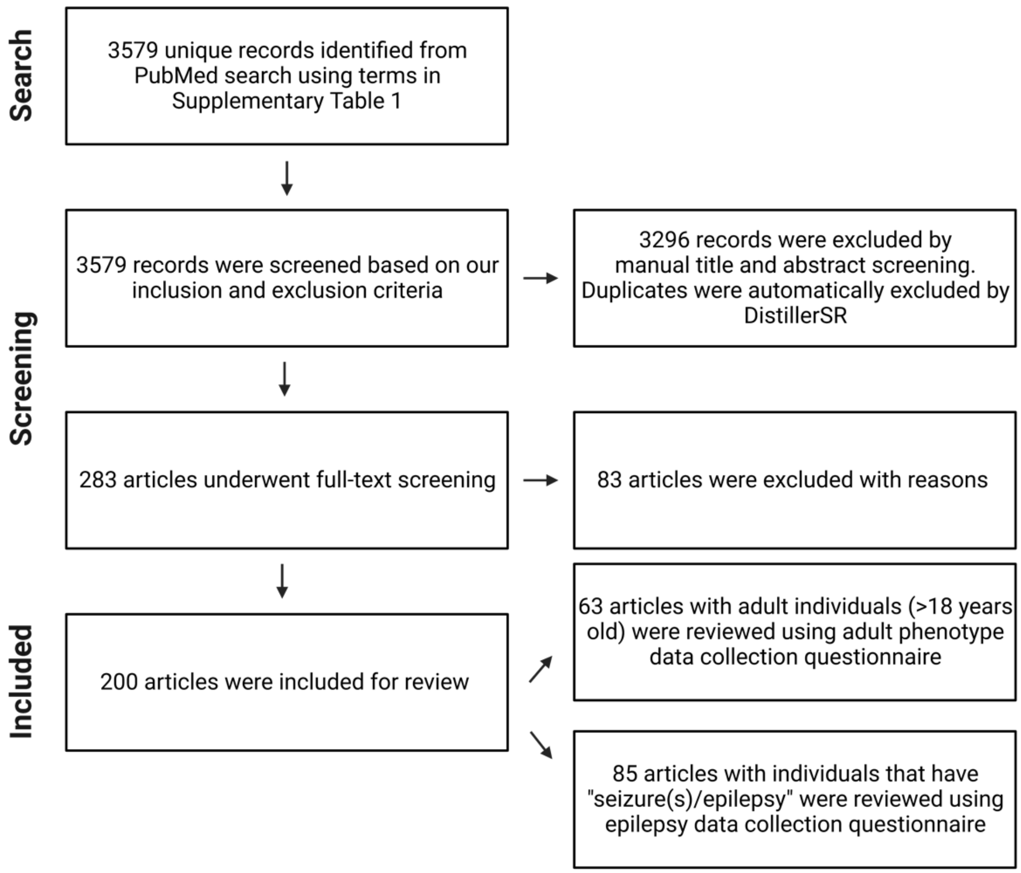

Supplementary Figure 2. PRISMA flow diagram for secondary search.
See main text for details.

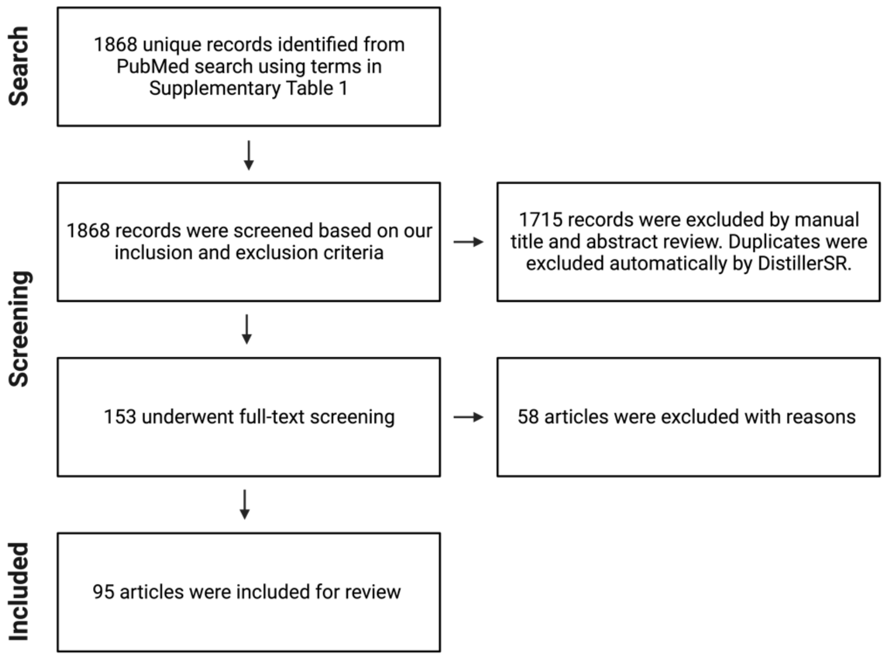

Supplementary Figure 3. Lollipop chart summarizing reporting of adulthood-related aspects of phenotype in n=63 reports of novel genetic disorders.
See main text for details.

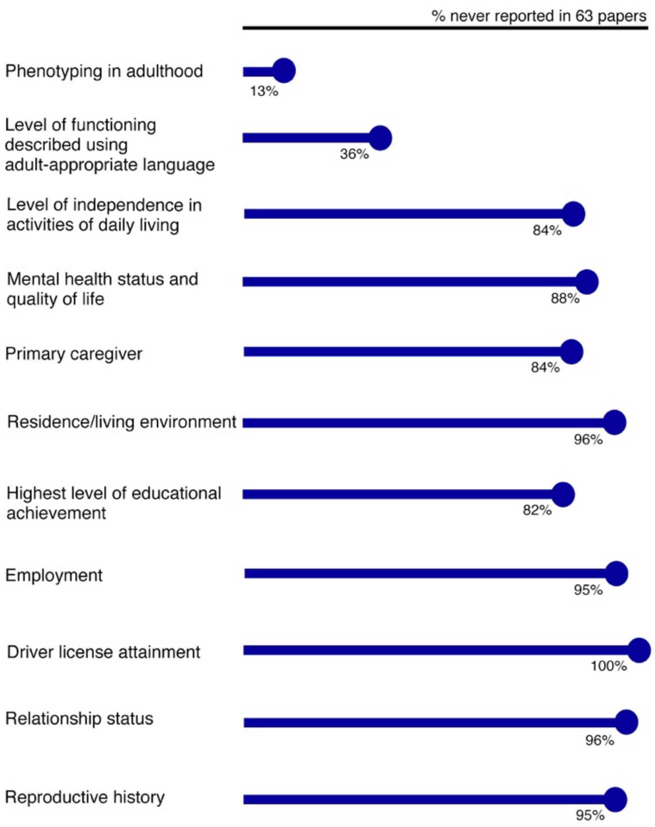

Supplementary Figure 4. Lollipop chart summarizing reporting of seizure/epilepsy-related aspects of phenotype in n=85 reports of novel genetic disorders.
See main text for details.

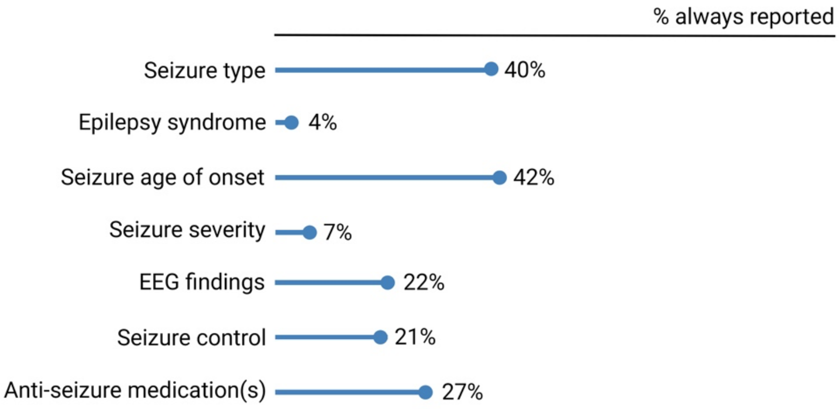

Supplementary Table 1. DistillerSR search details.

Search Type	Search Date	Search Terms
Initial reports	January 3, 2022	(Nature Genetics[journal] OR American Journal of Human Genetics[journal] OR Genome Medicine[journal] OR PloS Genetics[journal] OR Genetics in Medicine[journal] OR Human Molecular Genetics[journal] OR Journal of Medical Genetics[journal] OR American Journal of Medical Genetics Part A[journal] OR European Journal of Human Genetics[journal] OR European Journal of Medical Genetics[journal] OR Journal of Human Genetics[journal] OR Clinical Genetics[journal]) AND 2017:2021[dp] AND (new[title] OR novel[title] OR associated[title] OR cause[title] OR causes[title])
Follow-up reports	June 1, 2022	OTUD6B OR PPM1D OR ISCA1 OR PSMD12 OR PLAA OR ZNF462 OR YY1 OR NKX6-2 OR KCNQ5 OR WDR26 OR LIPT2 OR UBTF OR SCAPER OR CDK10 OR HIST1H4C OR C1QBP OR BPTF OR PPP3CA OR FDXR OR DHX30 OR CAMK2A OR CAMK2B OR H4C3 OR GPAA1 OR RAB11B

Supplementary Table 2. Publication details for the n=200 reports of novel genetic disorders included in this study.
See main text and Supplementary Figure 1 for details. There were no significant differences in the distribution of qualitative assessments of phenotype Domains by year or by journal (data not shown).

**Year of Publication**	**Number of Reports**
2017	24
2018	38
2019	57
2020	37
2021	44
**Journal**	
American Journal of Human Genetics	134
Clinical Genetics	4
European Journal of Human Genetics	9
Genetics in Medicine	19
Genome Medicine	1
Human Molecular Genetics	15
Journal of Medical Genetics	11
Nature Genetics	5
PLoS Genetics	2

Supplementary Table 3. Ages of research participants in 200 reports of novel genetic disorders included in this study.

Age range in years	Number of individuals (% of n=1856)
<2	203 (10.9%)
2-7	651 (35.1%)
8-17	622 (33.5%)
>17	263 (14.2%)
Not reported	117 (6.3%)

Supplementary Table 4. Phenotype extraction form data for Domains I-IV for the initial and follow-up reports.

	Initial Reports (n=200)	Follow-up Reports (n=95)
**DOMAIN I: DEVELOPMENT, COGNITION, AND MENTAL HEALTH**		
1. Are development and/or cognition issues mentioned as a feature? (e.g., global developmental delay / intellectual disability / encephalopathy/ cognitive impairment / motor impairment / social communication deficits)	Yes: 96.5%<br>No: 3.5%	Yes: 87.4%<br>No: 12.6%
a. Are qualifiers used? (e.g., mild, moderate, severe, profound)	Always: 42%<br>Sometimes: 40.4%<br>Never:17.6%	Always: 43.4%<br>Sometimes: 19.3%<br>Never: 37.3%
b. Are narrative descriptions of development and cognition provided?	Always: 21.2%<br>Sometimes: 53.9%<br>Never: 24.9%	Always: 61.4%<br>Sometimes: 12.1%<br>Never: 26.5%
c. Were formal assessments performed? (e.g., WISC, WAIS, Bayley)	Always: 0.5%<br>Sometimes: 37.5%<br>Never: 62%	Always: 19.3%<br>Sometimes: 16.8%<br>Never: 63.9%
2. Are behavioural and/or psychiatric issues mentioned as a feature?	Yes: 62.5%<br>No: 37.5%	Yes: 50%<br>No: 50%
a. Are details given regarding assessment, type, and severity?	Always: 4.8%<br>Sometimes: 75.2%<br>Never: 2%	Always: 13%<br>Sometimes: 13%<br>Never: 74%
b. Is age of onset specified?	Always: 6.4%<br>Sometimes: 24.8%<br>Never: 68.8%	Always: 6.5%<br>Sometimes: 2.2%<br>Never: 91.3%
c. Is treatment mentioned?	Always: 0.8%<br>Sometimes: 12.8%<br>Never: 86.4%	Always: 6.5%<br>Sometimes: 2.2%<br>Never: 91.3%
3. Is adaptive functioning or a relevant proxy/component described? (i.e., continence, hearing, vision,	Yes: 95%<br>No: 5%	Yes: 76.8%<br>No: 23.2%

technology dependence, communication)		
a. Is continence mentioned?	Always: 0%<br>Sometimes: 11.8%<br>Never: 88.2%	Always: 4.1%<br>Sometimes: 5.4%<br>Never: 90.5%
b. Is vision mentioned?	Always: 34.2%<br>Sometimes: 40.5%<br>Never: 25.3%	Always: 56.8%<br>Sometimes: 17.6%<br>Never: 25.7%
c. Is hearing mentioned?	Always: 22.1%<br>Sometimes: 38.4%<br>Never: 39.5%	Always: 21.6%<br>Sometimes: 16.2%<br>Never: 62.2%
d. Is communication style / ability mentioned?	Always: 33.2%<br>Sometimes: 57.9%<br>Never: 8.9%	Always: 48.6%<br>Sometimes: 21.6%<br>Never: 29.8%
4. Is adaptive functioning described in quantitative terms? (e.g., ABAS or Vineland)?	Always: 0%<br>Sometimes: 7.4%<br>Never: 92.6%	Always: 10.8%<br>Sometimes: 4.1%<br>Never: 85.1%
5. Is technology (assistive devices) use mentioned?	Yes: 56.6%<br>No: 43.3%	Yes: 33.3%<br>No: 66.7%
a. Is need for breathing tube mentioned?	Always: 0%<br>Sometimes: 17%<br>Never: 83%	Always: 3.8%<br>Sometimes: 15.4%<br>Never: 80.8%
b. Is need for wheelchair mentioned?	Always: 0%<br>Sometimes: 32%<br>Never: 68%	Always: 0%<br>Sometimes: 24%<br>Never: 76%
c. Is need for feeding tube mentioned?	Always: 8.4%<br>Sometimes: 74.8%<br>Never: 16.8%	Always: 24%<br>Sometimes: 48%<br>Never: 28%
6. Overall rating for [this domain]	Absent: 1.5%<br>Superficial/Deficient: 85.5%<br><br>Adequate: 12.0%<br>Strong: 1%	Absent: 5.3%<br>Superficial/Deficient: 76.8%<br><br>Adequate: 12.6%<br>Strong: 5.2%
**DOMAIN II: FEEDING AND GROWTH**		

7. Are "feeding difficulties" mentioned as a feature?	Always: 18%<br>Sometimes: 50%<br>Never: 32%	Always: 29.5%<br>Sometimes: 20%<br>Never: 50.5%
a. Are any additional qualifiers or contextual details provided at all (e.g., age at onset, severity, and/or necessary supports)?	Always: 8.2%<br>Sometimes: 74.6%<br>Never: 17.2%	Always: 18.8%<br>Sometimes: 12.5%<br>Never: 68.7%
8. Is growth mentioned?	Always: 59%<br>Sometimes: 33.5%<br>Never: 7.5%	Always: 69.5%<br>Sometimes: 2.1%<br>Never: 28.4%
a. Are the actual growth measurements and corresponding ages provided? (not just percentile ranges)	Always: 49.2%<br>Sometimes: 37%<br>Never: 13.5%	Always: 62.3%<br>Sometimes: 17.4%<br>Never: 20.3%
b. Are measurements provided for two or more time points (outside of birth parameters)?	Always: 1.1%<br>Sometimes: 4.9%<br>Never: 94%	Always: 7.3%<br>Sometimes: 5.8%<br>Never: 86.9%
9. Overall rating for [this domain]	Absent: 3%<br>Superficial/ Deficient: 87%<br>Adequate: 9%<br>Strong: 1 %	Absent: 24.2%<br>Superficial/ Deficient: 64.2%<br>Adequate: 10.5%<br>Strong: 1.1%
**DOMAIN III: MEDICATION USE AND TREATMENT HISTORY**		
10. Are there any aspects of the phenotype that suggest a possible role for medication(s)? (e.g., seizures, "behavioural problems", aggression, sleep disturbance, etc)	Yes: 86.5%<br>No: 13.5%	Yes: 66.3%<br>No: 33.7%
11. Are any of an individual's prescription medications listed?	Always: 8.5%<br>Sometimes: 38%<br>Never: 53.5%	Always: 20%<br>Sometimes: 10.5%<br>Never: 69.5%
12. Is the total number of prescribed medications per participant listed?	Always: 5.5%<br>Sometimes: 15.5%<br>Never: 79%	Always: 2.1%<br>Sometimes: 1.1%<br>Never: 96.8%
13. Are adverse drug events (or lack thereof) mentioned?	Always: 0%<br>Sometimes: 5%<br>Never: 95%	Always: 6.3%<br>Sometimes: 2.1%<br>Never: 91.6%
14. Are successful drug trials mentioned? (i.e., positive responses to medications)	Always: 4%<br>Sometimes: 25.5%<br>Never: 70.5%	Always: 15.8%<br>Sometimes: 9.5%<br>Never: 74.7%

15.	Are unsuccessful drug trials mentioned? (i.e., negative responses to medications)	Always: 2.5%<br>Sometimes: 18.5%<br>Never: 79%	Always: 15.8%<br>Sometimes: 8.4%<br>Never: 75.8%
16.	Were other treatments discussed? (e.g., dietary, surgical; excluding nutritional interventions indicated previously)	Yes: 48.5%<br>No: 51.5%	Yes: 37.9%<br>No: 62.1%
17.	Overall rating for [this domain]	Absent: 35.5%<br>Superficial /Deficient: 52.5%<br>Adequate: 11%<br>Strong: 1%	Absent: 48.4%<br>Superficial /Deficient: 32.6%<br>Adequate: 12.4%<br>Strong: 6.3%
**DOMAIN IV: PAIN, SLEEP, AND QUALITY OF LIFE**			
18.	Is the presence/absence of pain and neuro-irritability mentioned?	Always: 0%<br>Sometimes: 18%<br>Never: 82%	Always: 6.3%<br>Sometimes: 7.4%<br>Never: 86.3%
19.	Is quality of life characterized using an established scale/questionnaire/assessment?	Always: 0%<br>Sometimes: 2.5%<br>Never: 97.5%	Always: 0%<br>Sometimes: 0%<br>Never: 100%
20.	Is the presence/absence of sleep problems mentioned?	Always: 4%<br>Sometimes: 35%<br>Never: 61%	Always: 7.4%<br>Sometimes: 11.6%<br>Never: 81%
21.	Is mortality mentioned?	Yes: 27.5%<br>No: 72.5%	Yes: 14.7%<br>No: 85.3%
	a. Are age at death and cause of death described?	Always: 51.9%<br>Sometimes: 38.9%<br>Never: 9.2%	Always: 80%<br>Sometimes: 13.3%<br>Never: 6.7%
	b. Is palliative care involvement mentioned?	Yes: 1.9%<br>No: 98.1%	Yes: 0%<br>No: 100%
22.	Is the presence/absence of sialorrhea mentioned?	Always: 0%<br>Sometimes: 8.5%<br>Never: 91.5%	Always: 6.3%<br>Sometimes: 7.4%<br>Never: 86.3%
23.	Is the presence/absence of recurrent aspirations mentioned?	Always: 1%<br>Sometimes:12%<br>Never: 87%	Always: 2.1%<br>Sometimes:4.2%<br>Never: 93.7%
24.	Are the presence/absence of tone abnormalities mentioned? (e.g., hypotonia, hypertonia, dystonia)	Always: 59.5%<br>Sometimes: 28.5%<br>Never: 12%	Always: 47.4%<br>Sometimes: 15.8%<br>Never: 36.8%

25.	Overall rating for [this domain]	Absent: 6%<br>Superficial/Deficient: 92%<br>Adequate: 2%<br>Strong: 0%	Absent: 26.3%<br>Superficial/Deficient: 72.6%<br>Adequate: 1.1%<br>Strong: 0%

Supplementary Table 5. PMIDs of the n=295 papers reviewed in this study.

28007986	29276004	30335141	30905399	31495489	32413283	33442026	34145223
28130356	29290337	30343942	30929739	31509304	32430360	33473207	34180050
28135719	29300972	30343943	30941876	31564433	32442410	33506510	34211179
28343629	29304374	30359776	30976112	31585109	32527956	33513338	34230638
28343630	29304375	30364145	30976113	31587868	32543299	33522091	34244665
28356563	29388673	30388400	30982608	31607425	32593294	33531666	34346499
28388435	29394991	30388402	31006512	31630790	32600977	33632302	34354232
28413018	29427787	30401460	31031012	31735293	32627382	33638881	34355505
28513610	29432562	30401461	31034465	31785787	32652806	33675273	34379057
28575647	29478781	30421579	31036918	31838722	32694869	33711248	34385670
28575651	29483653	30455226	31069901	31883644	32707086	33742450	34411328
28669405	29560374	30481285	31079897	31916397	32721402	33743206	34413497
28686853	29576218	30487643	31079898	31924697	32730804	33782605	34419324
28692176	29656858	30500825	31079899	31929335	32738225	33796307	34585832
28757203	29656859	30503518	31089205	31931739	32779182	33811806	34587489
28777933	29656860	30517966	31092906	31949313	32820033	33824500	34618373
28794130	29726930	30526862	31124279	32004446	32822602	33847457	34703884
28837161	29758292	30526868	31147255	32004679	32875707	33864376	34707297
28866611	29767723	30549423	31155615	32030560	32885237	33909591	34715011
28886341	29808498	30561111	31178128	32031333	32891193	33909990	34729769
28920961	29907796	30580808	31192531	32092383	32924626	33909992	34804873
28934986	29961568	30595371	31204009	32109420	33026538	33938912	34906456
28940097	29961569	30595372	31230721	32152250	33073849	33963760	34906466
28942965	29979980	30609410	31256876	32181568	33110267	33975400	34906501
28942966	30057030	30612693	31278393	32197073	33159882	33979636	35016835
28942967	30057031	30633344	31303265	32197074	33186545	33991472	35027293
28965846	30105122	30639322	31322726	32197075	33217309	34003581	35080150
28969374	30122539	30661771	31327508	32214227	33232675	34003604	35172867
29040572	30214071	30661772	31327510	32220290	33245860	34010604	35198003
29100085	30224647	30679813	31353023	32220291	33276377	34020708	35202563
29100089	30250212	30723319	31353024	32246862	33308444	34022130	35377796
29100095	30254215	30755392	31361404	32275884	33344382	34050707	35430327
29106825	30269814	30795918	31363758	32307552	33348459	34055682	35503477
29130579	30290153	30824121	31402090	32330417	33355653	34077761	35583973
29158550	30290154	30827496	31422817	32338762	33369188	34102099	35620293
29251763	30293988	30827498	31422819	32341456	33417887	34114225	35627197
29267967	30323019	30905398	31439720	32356556	33420346	34143952	

Supplementary Table 6. Phenotype extraction form data for Domain V for the initial reports describing adults.

Question	Answers (n=63)
1. Are there any other adult individuals (age >=18 years old) who were referenced in this report as (likely) being affected with the genetic disease (e.g., there is mention of the variant being inherited from "parent with …", but no other details are provided and that parent is not participating in the study)?	Yes: 6.3%<br>No: 93.7%
2. Was/were the adult(s) phenotyped in adulthood?	Always: 85.7%<br>Sometimes: 1.6%<br>Never: 12.7%
3. Is there any description related to the adult's mental health status or quality of life?	Yes: 64.3%<br>No: 35.7%
4. Is the level of functioning described using adult-appropriate (e.g., not "global developmental delay") language?	Yes: 64.3%<br>No: 35.7%
5. Is highest level of educational achievement explicitly mentioned?	Always: 1.6%<br>Sometimes: 15.9%<br>Never: 82.5%
6. Is their residence/living environment mentioned (e.g., group home, residential care, supported housing, lives independently)?	Always: 1.6%<br>Sometimes: 3.2%<br>Never: 95.2%
7. Is their relationship status (past or present) mentioned?	Always: 1.6%<br>Sometimes: 1.6%<br>Never: 96.8%
8. Was their reproductive history described?	Always: 3.2%<br>Sometimes: 1.6%<br>Never: 95.2%
9. Is employment explicitly mentioned?	Always: 1.6%<br>Sometimes: 3.2%<br>Never: 95.2%
10. Is their level of independence in activities of daily living mentioned (e.g., dressing, eating, toileting, chores/work, etc.)?	Always: 6.4%<br>Sometimes: 9.5%<br>Never: 84.1%
11. Is driver license attainment mentioned?	Always: 0%<br>Sometimes: 0%

	Never: 100%
12. Is their primary caregiver mentioned / implied?	Always: 11.1%<br>Sometimes: 4.8%<br>Never: 84.1%

Supplementary Table 7. Phenotype extraction form data for Domain VI for the initial reports describing individuals with seizures/epilepsy.

Question	Answers (n=85)
1. Was epilepsy considered by the authors to be a major feature of the condition (e.g., epilepsy is mentioned in the title or abstract)?	Yes: 30.6%<br>No: 69.4%
2. Is seizure type(s) mentioned (at least at one timepoint)?	Always: 40%<br>Sometimes: 42.4%<br>Never: 17.6%
3. Is the epilepsy syndrome(s) mentioned (at least at one timepoint)?	Always: 3.5%<br>Sometimes: 14.1%<br>Never: 82.4%
4. Is age of onset of seizures mentioned?	Always: 42.4%<br>Sometimes: 34.1%<br>Never: 23.5%
5. Are EEG findings explicitly described?	Always: 22.4%<br>Sometimes: 43.5%<br>Never: 34.1%
6. Are any individuals mentioned who had sudden unexpected death in epilepsy, or otherwise died from an epilepsy-related complication?	Yes: 5.4%<br>No: 94.6%
7. Are current anti-seizure medications (or lack thereof) mentioned?	Always: 22.4%<br>Sometimes: 42.5%<br>Never: 34.1%
a. Is dosage mentioned?	Always: 0%<br>Sometimes: 8.5%<br>Never: 91.5%
b. Is there mention of anti-seizure treatments that were trialed and then discontinued for any participant?	Yes: 31.8%<br>No: 68.2%
c. Is this a variable that was clearly documented for all participants with seizures/epilepsy?	Yes: 33.3%<br>No: 66.7%
d. Are adverse drug events (or lack thereof) mentioned that relate to anti-seizure treatments, for any participant?	Yes: 4.7%<br>No: 95.3%

e. Is this a variable that was clearly documented for all participants with seizures/epilepsy?	Yes: 0%<br>No: 100%
8. Is there explicit mention for any participant of dietary interventions for seizures (e.g., ketogenic diet)?	Yes: 16.5%<br>No: 83.5%
a. Is this a variable that was clearly documented for all participants with seizures/epilepsy?	Yes: 21.4%<br>No: 78.6%
9. Is there explicit mention for any participant of surgical interventions for seizures (e.g., VNS insertion, epilepsy surgery)?	Yes: 7.1%<br>No: 92.9%
a. Is this a variable that was clearly documented for all participants with seizures/epilepsy?	Yes: 16.7%<br>No: 83.3%

Supplementary Table 8. Summary of the modified Delphi process used to formulate the PHELIX_General version 1.0 reporting checklist.

Stage	Date(s)	Total number of items discussed and ranked	Engagement
**1. Systematic review of the existing literature**	January 2022 – December 2022	Not applicable	Not applicable
**2. Electronic surveys**	January 2023 – April 2023	50	9 of 11
**3. Large group meeting**	May 18, 2023	35	Live meeting attendance: 6<br><br>Feedback after asynchronous viewing: 1
**4. Final review and approval of checklist items**	August 2023 – September 2023	See Table 1 for details	All listed co-authors

Supplementary Table 9. Expanded PHELIX_General (version 1.0) with examples and notes.

Domain	Recommendation Category	Recommendations	Examples/Notes
**1. Development and Cognition**	Strongly recommended	i. Standard and specific diagnostic term(s) for cognitive or developmental issue(s)<br><br>ii. Level of cognitive functioning or degree of developmental delay<br><br>iii. Age of attaining major milestones<br><br>iv. Quantitative results from psychometric testing OR explicit acknowledgement that these results were not available	PMID: 30609410<br>Refer to case description of family 1 in the article and supplementary file.<br><br>PMID: 30487643<br>Refer to case descriptions for individuals 1 and 3 in the article.
	Optional, but encouraged	v. Narrative summary describing progression / change in cognitive or development issue(s) over time.	
**2. Behaviour and Neuropsychiatric Conditions**	Strongly recommended	i. Standard and specific diagnostic term(s) for behavioural issues	PMID: 30487643<br>Refer to case description for individual 2
	Optional, but encouraged	ii. Reported functional impact of behavioural/psychiatric condition<br>iii. Age at diagnosis and/or age at first concern for behavioural issues<br>iv. Impact of treatments / interventions, as reported by individuals, families, and/or clinicians	

**3. Other Medical Conditions**	Strongly recommended	i.Major medical conditions<br><br>ii. Presence or absence of issues in the following areas (if potentially associated with the condition under study):<br><br>- Visual acuity and field of vision<br>- Hearing ability<br>- Speech/communication styles<br>- Continence/toileting<br>- Ambulation	PMID: 28692176<br>Refer to case descriptions of all patients in the article that consistently mention the absence of hearing and vision issues.
**4. Feeding Issues**	Strongly recommended	i. Functional impact of feeding issues<br><br>ii. Current feeding method (e.g., oral, gastrostomy tube)	PMID: 30401460<br>Refer to case report for individual 1 in the supplementary file.
	Optional, but encouraged	iii. Age at first concern for feeding issues<br><br>iv. Interventions and supports for feeding issues (e.g., feeding tube support)	
**5. Growth**	Strongly recommended	i. Birth growth measurements AND gestational age-corrected centiles<br><br>ii. Growth measurements (absolute values and z-scores) at two or more post-birth timepoints (where possible)	PMID: 28920961<br>Refer to patient report 1 in the supplementary file.
**6. Medication and Treatment History**	Optional, but encouraged	i. Details of efficacious treatment(s)<br><br>ii. Severe adverse events / reactions	PMID: 32220291<br>Refer to case report for individual 2 in the supplementary file that reports on medications trialed for seizures.
**7. Pain, Sleep, and Quality of Life**	Optional, but encouraged	i. Presence or absence of pain / neuroirritability<br><br>ii. Presence or absence of abnormal sleep patterns / sleep disturbance	PMID: 33632309<br><br>Refer to Table 6 as examples of

		iii. Qualitative description of proxies for quality of life, via patient and/or caregiver report<br><br>iv. Direct assessment of quality of life using established measure(s), via patient and/or caregiver report	reporting sleeping disorders<br><br>PMID: 36740581
**8. Indicators of adult functional outcome**	Strongly recommended	i. Age at which the adult was last seen/phenotyped<br><br>ii. Description of educational achievement<br><br>iii. Nature of any employment (past and/or present)	PMID: 25569435, 36729053
	Optional, but encouraged	iv. Relationship status (past and/or present)<br><br>v. Reproductive history	
**9.Other**	Strongly recommended	i. Confirmation of informed consent to participate in the research study and to include the above phenotype information, for each participant<br><br>ii. Distinguish between "not assessed" and "assessed and not present," for every aspect of a phenotype described in the report and for each participant<br><br>iii. Description of how phenotyping was performed (e.g., direct assessment by study team member(s), review of medical records, information provided on testing requisition), for each participant<br><br>iv. Use of phenotype ontologies (e.g., HPO, ICD-11) and reporting tools (e.g., Phenopackets Schema) to standardize reporting, for each participant<br><br>v. [For deceased participants] Cause of death	PMID: 34284219

Abbreviations: HPO, Human Phenotype Ontology; ICD-11, International Classification of Diseases 11th Revision

Supplementary Table 10. Expanded PHELIX_Epilepsy (version 1.0) reporting guideline with examples.

Domain	Recommendation Category	Recommended Information To Include In Epilepsy / Seizure Phenotype Reporting (If Applicable In Clinical Context)	Examples/Notes
**1. Epilepsy syndrome and severity**	Strongly recommended	i. Seizure type(s) (per ILAE)<br>ii. Age at seizure onset<br>iii. EEG findings (including age(s) at time of study)<br>iv. Epilepsy syndrome(s) (per ILAE)<br>v. Findings that support the diagnosis of the epilepsy syndrome(s) (e.g., specific EEG findings)<br>vi. Seizure frequency at last clinical assessment<br>vii. Qualifiers of overall epilepsy severity (e.g., severe; treatment-refractory)	PMID: 28942967<br>Refer to Table 1 and accompanying Results section
	Optional, but encouraged	viii. Clarification if EEG data were directly reviewed by members of the study team (versus only report details extracted from medical record)<br>ix. Number of seizures requiring hospitalization in specific timeframe (e.g., last year)	
**2. Pharmacological interventions**	Strongly recommended	i. Current and past medication name(s)<br>ii. Perceived impact on seizure control	PMID: 32220291<br>Refer to case report for individual 2 in the supplementary file that reports on medications trialed for seizures.
	Optional, but encouraged	iii. Dose<br>iv. Duration of treatment trial<br>v. Adverse effects/events due to the intervention	
**3. Non-pharmacological interventions**	Strongly Recommended	i. Intervention / procedure details (e.g., ketogenic diet, neurosurgery)<br>ii. Perceived impact on seizure control	PMID: 28942967<br>Refer to Table 1 and accompanying Results section

	Optional, but encouraged	iii. Adverse effects/events due to the intervention	
**4. Brain imaging findings**	Strongly Recommended	i. Brain imaging findings (including age(s) at time of study	PMID: 30323019 Refer to reports on patients 1 and 2 in the article.
	Optional, but encouraged	ii. Clarification if brain imaging data were directly reviewed by members of the study team (versus only report details extracted from medical record)	
**5. Other**	Optional, but encouraged	i. Narrative summary of the progression of the individual's seizure(s)/epilepsy phenotype over time<br>ii. Narrative summary of the progression of the individual's non-epilepsy phenotype over time (e.g., see PHELIX_General guidelines)	

Supplementary Table 11. Eight common problems with phenotype reporting observed by the study team.

Phenotype Domain	Common Problem
Development and cognition	• Using only "+" or "-" to describe the presence or absence of developmental delay and/or intellectual disability, and not providing further details regarding the nature or severity of those features.<br>• Reporting only on "global developmental delay", and not intellectual disability, for individuals older than 5 years of age.
Behaviour and neuropsychiatric conditions	• Using the term "behavioural issues" without additional details.
Feeding issues	• Using the term " feeding issues" without additional details.
Growth	• Not providing both absolute and centile/z-score measurements.
Medication and treatment history	• Not mentioning pertinent medication trials (e.g., no mention of anti-seizure medication responses in a cohort of individuals who mostly have epilepsy).
Adulthood phenotype elements	• Reporting only paediatrics-focused phenotypes for adult participants, including no mentions of adult functioning.
Other	• Not distinguishing situations where a phenotype was assessed and absent from situations where a phenotype was not assessed.

Supplementary Information

IPCHiP Executive Committee members: Nancy C. Andrews[23], Alan H. Beggs[8,23,24], John Christodoulou[10,18], J. Helen Cross[12,25], Christian R. Marshall[26,27,28], Kathryn N. North[10,18], Stephen W. Scherer[2,4,27], Neil J. Sebire[25], Piotr Sliz[23,29]

[2]Program in Genetics & Genome Biology, SickKids Research Institute, Toronto, Canada

[4]Department of Molecular Genetics, University of Toronto, Toronto, Canada

[8]Department of Pediatrics, Harvard Medical School, Boston, MA, USA

[10]Murdoch Children's Research Institute, Melbourne, Australia

[12]Department of Neurology, Great Ormond Street Hospital, London, UK

[18]Department of Paediatrics, University of Melbourne, Melbourne, Australia

[23]Department of Pediatrics, Boston Children's Hospital, Boston, MA, USA

[24]The Manton Center for Orphan Disease Research, Boston Children's Hospital, Boston, MA, USA

[25]UCL Great Ormond Street Institute of Child Health, London, UK

[26]Division of Genome Diagnostics, Hospital for Sick Children, Toronto, Canada

[27]The Centre for Applied Genomics, SickKids Research Institute, Toronto, Canada

[28]Department of Laboratory Medicine and Pathobiology, University of Toronto, Toronto, Canada

[29]Department of Biological Chemistry and Molecular Pharmacology, Harvard Medical School, Boston, MA, USA
