# Supplementary material for: Consensus reporting guidelines to address gaps in descriptions of ultra-rare genetic conditions
Source: NPJ Genom Med. 2024 Apr 6;9:27. doi: 10.1038/s41525-024-00408-w (PMC10998895; doi:10.1038/s41525-024-00408-w)
Supplement: Supplementary file 2 — REPORTING SUMMARY [file 41525_2024_408_MOESM2_ESM.pdf]

nature portfolio

Corresponding author(s): Danielle Baribeau, Gregory Costain

Last updated by author(s): Jan 4, 2024

Reporting Summary

Nature Portfolio wishes to improve the reproducibility of the work that we publish. This form provides structure for consistency and transparency in reporting. For further information on Nature Portfolio policies, see our Editorial Policies and the Editorial Policy Checklist.

Statistics

For all statistical analyses, confirm that the following items are present in the figure legend, table legend, main text, or Methods section.

n/a	Confirmed	
☐	☒	The exact sample size (*n*) for each experimental group/condition, given as a discrete number and unit of measurement
☒	☐	A statement on whether measurements were taken from distinct samples or whether the same sample was measured repeatedly
☒	☐	The statistical test(s) used AND whether they are one- or two-sided<br>*Only common tests should be described solely by name; describe more complex techniques in the Methods section.*
☒	☐	A description of all covariates tested
☒	☐	A description of any assumptions or corrections, such as tests of normality and adjustment for multiple comparisons
☒	☐	A full description of the statistical parameters including central tendency (e.g. means) or other basic estimates (e.g. regression coefficient) AND variation (e.g. standard deviation) or associated estimates of uncertainty (e.g. confidence intervals)
☒	☐	For null hypothesis testing, the test statistic (e.g. *F*, *t*, *r*) with confidence intervals, effect sizes, degrees of freedom and *P* value noted<br>*Give P values as exact values whenever suitable.*
☒	☐	For Bayesian analysis, information on the choice of priors and Markov chain Monte Carlo settings
☒	☐	For hierarchical and complex designs, identification of the appropriate level for tests and full reporting of outcomes
☒	☐	Estimates of effect sizes (e.g. Cohen's *d*, Pearson's *r*), indicating how they were calculated

Our web collection on statistics for biologists contains articles on many of the points above.

Software and code

Policy information about availability of computer code

Data collection	N/A
Data analysis	N/A

For manuscripts utilizing custom algorithms or software that are central to the research but not yet described in published literature, software must be made available to editors and reviewers. We strongly encourage code deposition in a community repository (e.g. GitHub). See the Nature Portfolio guidelines for submitting code & software for further information.

Data

Policy information about availability of data

All manuscripts must include a data availability statement. This statement should provide the following information, where applicable:

- Accession codes, unique identifiers, or web links for publicly available datasets
- A description of any restrictions on data availability
- For clinical datasets or third party data, please ensure that the statement adheres to our policy

The datasets analysed during the current study are available from the corresponding authors on reasonable request.

Research involving human participants, their data, or biological material

Policy information about studies with human participants or human data. See also policy information about sex, gender (identity/presentation), and sexual orientation and race, ethnicity and racism.

Reporting on sex and gender	N/A
Reporting on race, ethnicity, or other socially relevant groupings	N/A
Population characteristics	N/A
Recruitment	N/A
Ethics oversight	N/A

Note that full information on the approval of the study protocol must also be provided in the manuscript.

Field-specific reporting

Please select the one below that is the best fit for your research. If you are not sure, read the appropriate sections before making your selection.

☒ Life sciences ☐ Behavioural & social sciences ☐ Ecological, evolutionary & environmental sciences

For a reference copy of the document with all sections, see nature.com/documents/nr-reporting-summary-flat.pdf

Life sciences study design

All studies must disclose on these points even when the disclosure is negative.

Sample size	We identified all first reports of novel genetic conditions discovered through genotype-driven ascertainment that result in multisystem and/or neurodevelopmental phenotypes, which were published in one of ten genetics journals that are known for publishing novel reports of ultra-rare genetic conditions (American Journal of Human Genetics, American Journal of Medical Genetics Part A, Clinical Genetics, European Journal of Human Genetics, Genetics in Medicine, Genome Medicine, Human Molecular Genetics, Journal of Medical Genetics, Nature Genetics, PLoS Genetics) during a 5-year period (1 January 2017 - 31 December 2021). We selected this period because of the rise to prominence of “gene matchmaker” tools in the mid- to late-2010s, and to ensure feasibility of the systematic data extraction.
Data exclusions	The search executed on January 3, 2022, identified 3,243 articles (Supplementary Table 1; Supplementary Figure 1). Exclusion criteria were: (i) prenatal or neonatal lethal phenotype, (ii) case report or description of a single family, (iii) new gene for a known clinical syndrome (e.g., Joubert syndrome, Noonan syndrome), (iv) chromosome disorder with non-recurrent breakpoints that did not definitively implicate a specific gene, (v) potential genotype-phenotype expansion rather than a novel disorder. We also excluded large-scale gene discovery efforts in populations with common complex diseases and/or clinical testing laboratory cohort studies, where the a priori expectation for detailed phenotype descriptions was low. After both title and abstract screening and full-text review stages, n=200 reports describing 199 distinct monogenic conditions met the inclusion criteria (two reports of a novel condition were published at the same time; Supplementary Figure 1). For the subset of 25 genetic conditions first described in 2017, we performed an additional search using DistillerSR on June 1, 2022 (Supplementary Table 1) to identify subsequent published case descriptions in any journal (total n=95; Supplementary Figure 2). Reference review and additional internet searching did not identify any other “follow-up” reports.
Replication	N/A
Randomization	N/A
Blinding	N/A

Reporting for specific materials, systems and methods

We require information from authors about some types of materials, experimental systems and methods used in many studies. Here, indicate whether each material, system or method listed is relevant to your study. If you are not sure if a list item applies to your research, read the appropriate section before selecting a response.

Materials & experimental systems

n/a	Involved in the study
☒	☐ Antibodies
☒	☐ Eukaryotic cell lines
☒	☐ Palaeontology and archaeology
☒	☐ Animals and other organisms
☒	☐ Clinical data
☒	☐ Dual use research of concern
☒	☐ Plants

Methods

n/a	Involved in the study
☒	☐ ChIP-seq
☒	☐ Flow cytometry
☒	☐ MRI-based neuroimaging

Plants

Seed stocks	N/A
Novel plant genotypes	N/A
Authentication	N/A
